# Cyr61/CCN1 Displays High-Affinity Binding to the Somatomedin B^ 1–44^ Domain of Vitronectin

**DOI:** 10.1371/journal.pone.0009356

**Published:** 2010-02-26

**Authors:** Ivo M. B. Francischetti, Michalis Kotsyfakis, John F. Andersen, Jan Lukszo

**Affiliations:** 1 Section of Vector Biology, Laboratory of Malaria and Vector Research, Research Technologies Branch, National Institute of Allergy and Infectious Diseases, National Institutes of Health, Bethesda, Maryland, United States of America; 2 Peptide Synthesis and Analysis Laboratory, Research Technologies Branch, National Institute of Allergy and Infectious Diseases, National Institutes of Health, Bethesda, Maryland, United States of America; 3 Institute of Parasitology, Biology Centre, National Academy of Sciences of Czech Republic, Ceske Budejovice, Czech Republic; University of Nebraska Medical Center, United States of America

## Abstract

**Background:**

Cyr61 is a member of the CCN (Cyr61, connective tissue growth, NOV) family of extracellular-associated (matricellular) proteins that present four distinct functional modules, namely insulin-like growth factor binding protein (IGFBP), von Willebrand factor type C (vWF), thrombospondin type 1 (TSP), and C-terminal growth factor cysteine knot (CT) domain. While heparin sulphate proteoglycans reportedly mediate the interaction of Cyr61 with the matrix and cell surface, the role of other extracellular associated proteins has not been revealed.

**Methods and Findings:**

In this report, surface plasmon resonance (SPR) experiments and solid-phase binding assays demonstrate that recombinant Cyr61 interacts with immobilized monomeric or multimeric vitronectin (VTNC) with *K_D_* in the nanomolar range. Notably, the binding site for Cyr61 was identified as the somatomedin B domain (SMTB ^1–44^) of VTNC, which mediates its interaction with PAI-1, uPAR, and integrin αvβ3. Accordingly, PAI-1 outcompetes Cyr61 for binding to immobilized SMTB ^1–44^, and Cyr61 attenuates uPAR-mediated U937 adhesion to VTNC. In contrast, isothermal titration calorimetry shows that Cyr61 does not display high-affinity binding for SMTB ^1-44^ in solution. Nevertheless, competitive ELISA revealed that multimeric VTNC, heat-modified monomeric VTNC, or SMTB ^1–44^ at high concentrations attenuate Cyr61 binding to immobilized VTNC, while monomeric VTNC was ineffective. Therefore, immobilization of VTNC exposes cryptic epitopes that recognize Cyr61 with high affinity, as reported for a number of antibodies, β-endorphin, and other molecules.

**Conclusions:**

The finding that Cyr61 interacts with the SMTB ^1–44^ domain suggests that VTNC represent a point of anchorage for CCN family members to the matrix. Results are discussed in the context of the role of CCN and VTNC in matrix biology and angiogenesis.

## Introduction

The Cyr61/CCN1 gene encodes a matricellular protein that belongs to the CCN family comprising CCN1/Cyr61; CCN2/CTGF (connective tissue growth factor); CCN3/NOV (nephroblastoma overexpressed); and CCN4-6/WISP1–3 (Wnt-inducible secreted proteins) [1]–[3]. Members of the CCN family have emerged as dynamically expressed, extracellular matrix-associated proteins that play critical roles in cardiovascular and skeletal development, injury repair, fibrotic diseases, and cancer. CCNs typically comprise four conserved cysteine-rich modular domains, with an N-terminal secretory peptide followed by four conserved domains with sequence homologies to insulin-like growth factor binding proteins (IGFBPs), von Willebrand factor type C repeat (vWC), thrombospondin type I repeat (TSP), and a carboxyl-terminal (CT) domain that contains a cysteine knot motif [1]–[3]. A series of ligands, including heparin sulphate proteoglycans (HSPG), growth factors, and integrins, reportedly interact with different domains of CCN members—these interactions may contribute to their unique activity and functions [1]–[3].

Similarly to CCN family members, the IGFBP family members are multidomain proteins with distinct cysteine-rich N- and C-terminal domains linked by a variable linker region. The family consists of six IGFBPs (1–6) that bind insulin growth factors (IGFs) with high affinity (dissociation constant [*K*_D_] 0.1 nM) to control their transport, localization, and metabolic breakdown [4]. Interestingly, the IGFBP domain of the CCN proteins shares strong sequence similarity to the N-terminal domain of traditional IGFBPs; this similarity has resulted in some CCN proteins being classified as additional IGFBPs or as IGFBP-related proteins [4]. Despite the similarity with the N-terminal domains, the CCN proteins bind IGF poorly, in the order of 100-fold lower affinity than the traditional IGFBPs. Recent evidence also indicates that some IGFBPs have actions that are IGF independent. For example, IGFBP-5 promotes cell growth and osteocalcin expression in IGF-I knockout mice [4]–[6] and displays high-affinity binding to vitronectin (VTNC) in an insulin growth factor-I (IGF-I) independent manner [7].

VTNC belongs to a group of adhesive glycoproteins that play key roles in the attachment of cells to their surrounding extracellular matrix (ECM) [8]–[11]. It plays a significant role in a number of physiologic processes such as cell adhesion, cell migration, wound healing, and regulation of the plasminogen activation (PA) system [12], [13]. In plasma, VTNC exists in a native, monomeric conformation, while during tissue injury or tumor growth, VTNC is recruited from the plasma into the extracellular matrix and assumes an active conformation through multimerization [14]. Many of the biologic functions of VTNC are mediated through interaction of the N-terminus (1–44 amino acids) somatomedin B domain (SMTB ^1–44^) of VTNC with a wide variety of ligands [15]–[22]. For example, it promotes cell attachment, spreading, and migration through interaction with cellular receptor integrins such as α_V_β_3_ and α_V_β_5_ [9]. VTNC also contributes to cell adhesion by binding to urokinase-type plasminogen activator receptor (uPAR) on the cell surface [18], [23], [24]. Further, VTNC interacts with high affinity with type 1 plasminogen activator inhibitor (PAI-1), the primary serine protease inhibitor (serpin) of both tissue- and urokinase-type plasminogen activators, leading to attenuation of fibrinolysis [12], [13].

In this paper, we hypothesize that members of the CCN family of proteins might interact with VTNC. Our *in vitro* results demonstrate that recombinant Cyr61 interacts with high affinity to immobilized multimeric or monomeric VTNC. Further, the binding site for Cyr61 was identified as the SMTB ^1–44^ domain of VTNC. Our results suggest that VTNC operates as an anchor for Cyr61, possibly modulating its biologic functions.

## Results

### Identification of Cyr61 as a Ligand for VTNC

**Figure 1:**
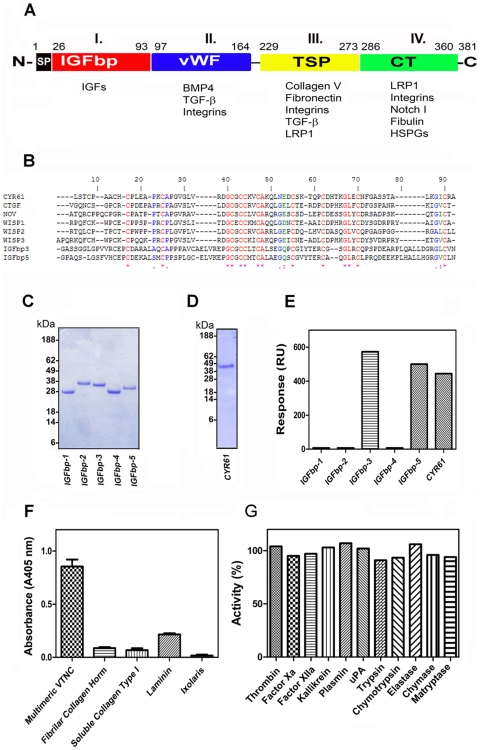
Cyr61 binds to VTNC. (**A**) Structure of CCN family members. CCN1 (Cyr61, represented in the figure), CCN2 (CTGF), CCN3 (NOV), CCN4 (WISP-1), CCN5 (WISP-2), and CCN6 (WISP-3) have a shared structure consisting of a secretory signal peptide (SP), an IGFBP domain (Module I), a von Willebrand type C domain (vWC, Module II), a thrombospondin-1 domain (TSP, Module III) and a cysteine knot (CT, Module IV) domain. Domains are linked by hinge regions susceptible to protease cleavage. Modified from reference [3]. (**B**) Clustal alignment of CCN family members with IGFBP-3 and -5 indicates several conserved residues (*). (**C**) and (**D**) SDS-PAGE for IGFBP-1 to -5, and Cyr61, respectively. Two µg of proteins were loaded in a 4–12% NU-PAGE gel, which was Coomassie blue stained. (**E**) SPR experiments: proteins (100 nM) were used as analytes for immobilized VTNC. Binding levels at stability was used as a value for comparison. RU, resonance units. (**F**) Specificity of Cyr61 (30 nM) tested by solid-phase binding assays: proteins were immobilized as the following densities/well: VTNC (25 ng), fibrilar collagen (50 ng), ixolaris (75 ng), soluble collagen and laminin (100 ng), followed by incubation with Cyr61 (30 nM). Bound-Cyr61 was identified using anti-Cyr61 monoclonal antibody. (**G**) Cyr61 does not inhibit enzyme activity. Cyr61 (300 nM) was incubated with different enzymes and fluorogenic substrate hydrolysis was followed as indicated in Materials and Methods. SEM are not depicted but were less than 5% (n = 3).

The CCN family of proteins is composed of six members, including Cyr61, CTGF, NOV, and WISP-1, -2, and -3. Figure 1A shows that they typically present four distinct functional modules, namely insulin-like growth factor (IGFBP), von Willebrand factor type C (vWF), thrombospondin type 1 (TSP), and C-terminal growth factor cysteine knot (CT) domain. Each of the four modules is thought to act both independently and interdependently; the ligands identified for each domain are specified in Figure 1A [1]–[3]. Figure 1B shows that the N-terminus of Cyr61 and other CCN family members display high sequence similarity to the IGFBP-3 and -5, which reportedly bind to VTNC with high affinity [7]. To verify whether IGFBPs and Cyr61 binds to VTNC, recombinant proteins were first assessed by SDS-PAGE to confirm correct molecular weight and purity (Figure 1, C and D). Cyr61 biologic activity has been determined by dose-dependent stimulation of the proliferation of 3T3 cells with a corresponding effective dose 50% (ED_50_) of 2–3 µg/ml according to the manufacturer (Peprotech Inc. certificate of analysis).

Multimeric VTNC was then immobilized (2851.4 resonance units [RU]) in a CM5 chip for screening assays and different ligands tested for binding using surface plasmon resonance (SPR). Figure 1E shows that only IGFBP-3 and -5 (100 nM) interact with VTNC as reported [7], and the same was observed for Cyr61, while IGFBP-1, -2, and 4 were ineffective. To verify binding and specificity by a second technique, solid-phase binding assays were optimized as described in Materials and Methods. Figure 1F demonstrates that Cyr61 binds to multimeric VTNC, which was detectable at levels of VTNC as low as 5 ng/well (not shown). Cyr61 also reproducibly interacted with other matrix proteins such as collagen and laminin, although at 15–20% of the value obtained for VTNC. Cyr61 binding to unrelated immobilized proteins such as ixolaris (a recombinant tissue factor pathway inhibitor from tick saliva) [25], which served as a control, was near basal levels.

An important control for the experiments described here has been to investigate the specificity of Cyr61 also by checking its effects using a series of enzymes that may play a role in tissue homeostasis. Figure 1G demonstrates that Cyr61 displays no inhibitory properties toward a panel of coagulation factors including thrombin, Factor Xa, Factor XIIa, and kallikrein. It is also devoid of inhibitory activity for uPA, trypsin, chymotrypsin, and other enzymes involved in inflammation such as elastase, chymase, and matryptase [26]. These results indicate that our preparation of Cyr61 binds to VTNC with high affinity, although it may also interact with other matrix proteins such as collagen and laminin.

### Cyr61 Is a High-Affinity Ligand for Immobilized VTNC

**Figure 2:**
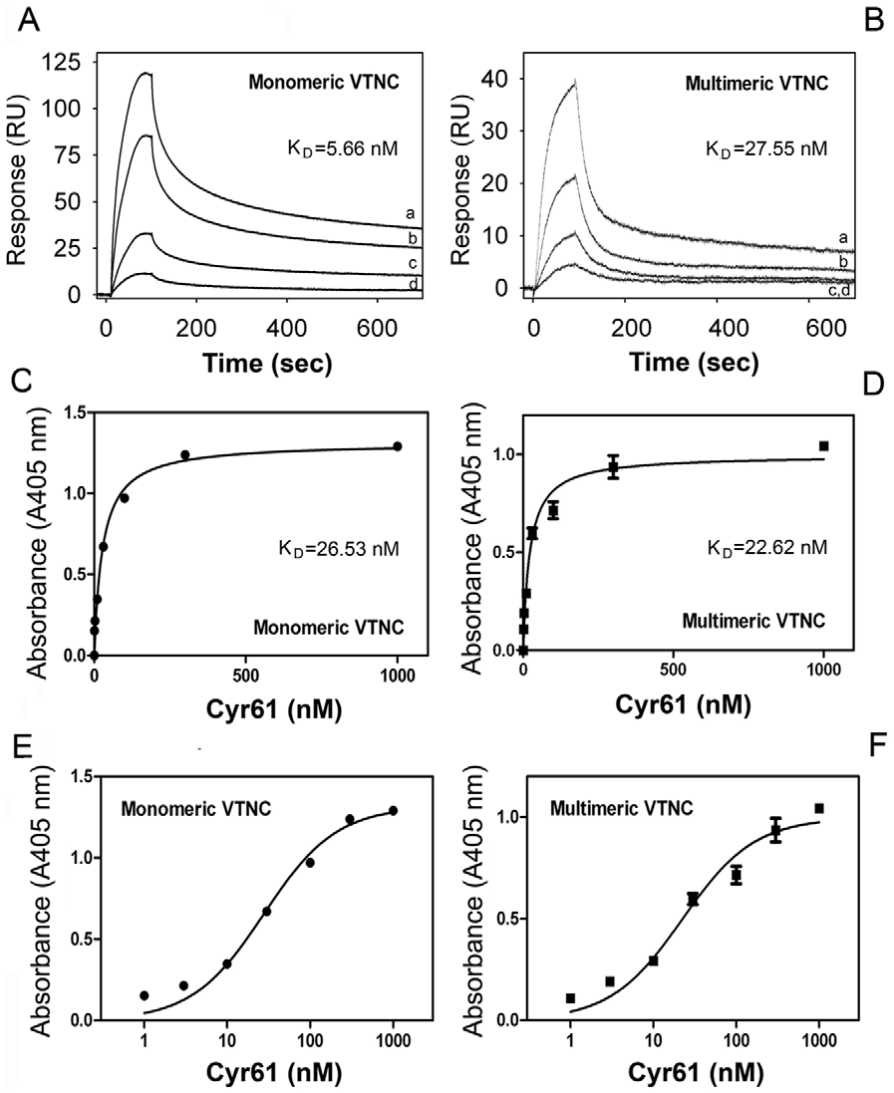
Cyr61 displays high-affinity binding to immobilized VTNC. (**A**) Sensorgrams for Cyr61 (in nM: *a*, 40; *b*, 20; *c*, 5; *d*, 2.5) binding to immobilized monomeric VTNC. (**B**) Sensorgrams show Cyr61 (in nM: *a*, 50; *b*, 25, *c*, 12.5; *d*, 6.25) binding to multimeric VTNC. Data were fitted using global two-state binding model. RU, resonance units. (**C**) and (**D**) solid-phase-binding assay for Cyr61 (0–1 µM) interaction with immobilized monomeric or multimeric VTNC, respectively. Binding was estimated with anti-Cyr61 monoclonal antibody followed by alkaline-phosphatase labeled anti-mouse secondary antibody and appropriate substrate as described in Materials and Methods. (**E**) and (**F**) Semi log-transformation of the data depicted in (**C**) and (**D**), respectively. The (apparent) *K_D_* values for Cyr61/VTNC interactions were calculated by nonlinear regression analysis of the binding data according to the Langmuir isotherm equation. All treatments were performed in quadruplicate or quintuplicate (n = 3).

To further characterize Cyr61 binding to VTNC, kinetics were performed using SPR experiments that detect real-time bimolecular interactions. For this set of experiments, VTNC was immobilized at low levels (∼600 RU) to avoid mass transfer effects [27]. An appropriate equation to fit the experimental results was chosen based on the residuals and calculated χ^2^ for each interaction. Sensorgrams collected for Cyr61 interaction with monomeric (Figure 2A) or multimeric (Figure 2B) VTNC display biphasic kinetics that fit best to a two-state reaction (conformational change) mechanism with two on- and off-rate constants yielding *K_D_* 5.66 and 27.55 nM, respectively. SPR experiments also suggest that Cyr61:VTNC complex formation displays a more complex binding mechanism and involves one binding site with a two-step reaction where an encounter complex, (Cyr61:VTNC)^*^, is observed before reaching the final complex state. Table 1 summarizes the kinetic findings.

**Table 1:** Kinetics of Cyr61 interactions with VTNC and SMTB ^1–44^.

	*ka1* (M^−1^s^−1^)	*kd1* (s^−1^)	*ka2* (s^−1^)	*kd2* (s^−1^)	*K_D_* (nM)
Monomeric VTNC	5.678×10^5^	0.02454	0.007829	0.001180	**5.66**
Multimeric VTNC	1.043×10^5^	0.06954	0.002555	0.000110	**27.55**
SMTB ^1–44^	1.273×10^5^	0.02501	0.01094	0.000592	**10.09**

**Figure 3:**
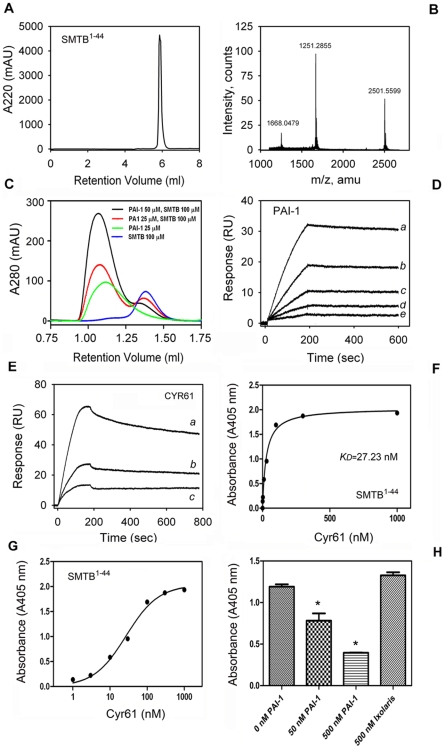
Cyr61 displays high-affinity binding to SMTB ^1–-44^ domain. (**A**) SMTB ^1–44^ was chemically synthesized and purified by reverse-phase chromatography. (**B**) Mass spectrometry for the synthetic peptide shows the expected mass for SMTB ^1–44^. (**C**) Gel-filtration chromatography shows complex formation between PAI-1 and SMTB ^1–44^ was performed as in Materials and Methods. (**D**) SPR experiments. Sensorgrams shows PAI-1 (in nM: *a*, 1.8; *b*, 0.9; *c*, 0.45; *d*, 0.225; *e*, 0.11) binding to immobilized SMTB ^1–44^ domain. (**E**) Sensorgrams show Cyr61 (in nM: *a*, 12.5; *b*, 6.25; *c*, 3.1) binding to the SMTB ^1–44^ domain. Data were fitted using global two-state binding model. RU, resonance units. (**F**) Solid-phase binding assay. Cyr61 (0–1 µM) was incubated with immobilized SMTB ^1–44^ and binding estimated with anti-Cyr61 monoclonal antibody, followed by alkaline phosphatase-labeled anti-mouse secondary antibody and appropriate substrate as described in Materials and Methods. (**G**) Semi-log transformation of the results presented in (**F**). The (apparent) *K_D_* values for Cyr61/ SMTB ^1–44^ interactions were calculated by nonlinear regression analysis of the binding data according to the Langmuir isotherm equation. (**H**) Competition experiments. Cyr61 (30 nM) was incubated with 0, 50, and 500 nM PAI-1, or 500 nM ixolaris. Mixtures were added to SMTB ^1–44^-coated wells and incubated for 90 minutes. SMTB ^1–44^-bound Cyr61 was estimated as in (**E**). Experiments were performed in quadruplicate or quintuplicate (n = 3).

Responses were obtained by injecting Cyr61 over immobilized VTNC or SMTB ^1–44^ for 90 seconds at a flow rate of 30 µl/minute. Data fitting using two-site binding model (conformational change). Kinetic values obtained from the sensorgrams are presented in Figure 2, C and D, and in Figure 3E.

Figure 2, C and D, respectively show the results for Cyr61 interaction with immobilized monomeric or multimeric VTNC estimated by solid-phase binding assays. Binding occurs in a dose-dependent manner, is saturable, and has an apparent (app) *K_D_* 26.53±5.31 nM for monomeric VTNC and 22.62±4.73 nM for multimeric VTNC. Data fitted with Langmuir equation for one binding site yield a |^2^ value (0.8927) comparable to two binding sites (0.9189). In addition, app *K_D_* value calculated by Langmuir equation is in close agreement with the *K_D_* calculated from the SPR data, which were fitted using a two-state model that takes into account one site (Table 1). Figure 2, E and F, respectively show semi-log transformation of data presented in Figure 2, C and D, which display a pattern suggestive of multiple binding sites or alternatively, due to heterogeneity in Cyr61 preparation or in immobilization of VTNC. In some experiments, Cyr61 interaction with multimeric VTNC was performed in the presence of TBS 0.4% Tween supplemented with 0.5% gelatin to exclude nonspecific interaction, and very similar results were obtained when compared to TBS 0.4% Tween only (app *K_D_* 36.54±9.6 nM).

### The SMTB ^1–44^ Domain Is a High-Affinity Binding Site for Cyr61

The SMTB ^1–44^ domain of VTNC comprises the first 44 amino acids of the protein and mediates its interaction with PAI-1, uPAR, and integrin αvβ3 [15]–[20]. In an attempt to verify whether Cyr61 binds to SMTB ^1–44^ domain, the peptide was synthesized, refolded, and purified to homogeneity as shown in Figure 3A. Mass spectrometry analysis of the fraction containing the SMTB ^1–44^ domain indicates a molecular mass of 5,003.00 Da., which is in agreement with the theoretical mass of 5,003.5 Da. for the peptide with eight oxidized cysteines (Figure 3B). A series of control experiments were performed to demonstrate that SMTB ^1–44^ was functionally folded. In the first set, gel-filtration chromatography was employed in an attempt to demonstrate PAI-1-SMTB ^1–44^ interactions in solution. Figure 3C shows that SMTB ^1–44^ applied to the column elutes at 1.38 ml (blue line), while PAI-1 elutes at 1.11 ml (green line). As the concentration of PAI-1 increases in a mixture containing a fixed concentration of SMTB ^1–44^, the peak corresponding to SMTB ^1–44^ decreases, and a peak corresponding to PAI-1-SMTB^1–44^ complex formation elutes at 1.06 ml (red and blacks lines).

**Figure 4:**
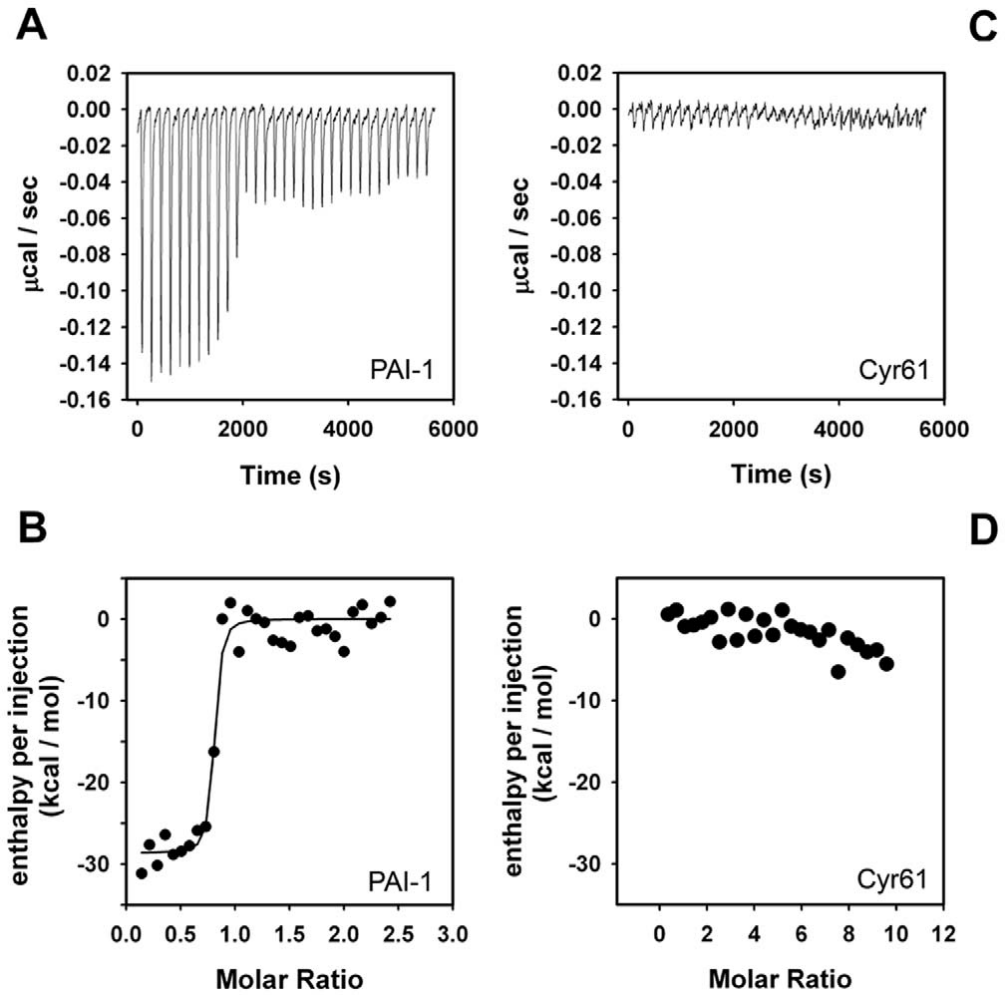
Cyr61 does not interact with SMTB ^1–44^ domain in solution. (**A**) Isothermal titration calorimetry for stable mutant PAI-1 interaction with SMTB ^1–44^. Base line-adjusted heats per injection of SMTB ^1–44^ (10 µM) into PAI-1 (1.0 µM). (**B**) Molar enthalpies per injection for PAI-1 interaction with SMTB ^1–44^. Filled circles, measured enthalpies; solid line, fit of experimental data to a single-site binding model. Thermodynamic parameters: Δ*H* = −24±1.34 kcal/mol. (**C**) Base line-adjusted heats per injection of SMTB ^1–44^ (10 µM) into Cyr61 (1.0 µM). (**D**) Molar enthalpies per injection for SMTB ^1–44^ interaction with Cyr61 where no heat exchange is observable. Filled circles, measured enthalpies.

In a second set of control experiments, the SMTB ^1–44^-PAI-1 interaction was analyzed by SPR where SMTB ^1–44^ was immobilized by amine coupling and PAI-1 served as the analyte [15]–[20]. Figure 3D shows that PAI-1 binds to SMTB ^1–44^ with a *K_D_* in the pM range (∼40 pM; Table 2) as previously reported [15]. Together, these controls validated the use of synthetic SMTB ^1–44^ for additional experimentation. Accordingly, Figure 4E illustrates sensorgrams for Cyr61 interaction with SMTB ^1–44^. Data were better described as a two-state conformational change, which yields a *K_D_* of ∼10 nM. In contrast, binding was not observed when ixolaris was used as the control analyte (data not shown).

**Table 2:** Kinetics of PAI-1 interactions with SMTB ^1–44^.

	*ka1* (M^−1^s^−1^)	*kd1* (s^−1^)	*K_D_* (pM)
SMTB ^1–44^	2.312×10^6^	0.00010	**43.25**

Responses were obtained by injecting stable mutant PAI-1 over immobilized SMTB ^1–44^ for 90 seconds at a flow rate of 30 µl/minute. Data were derived from *Ka1* and *Kd1* and fitted using the Langmuir (1∶1 binding) equation. Kinetic values obtained from the sensorgrams presented in Figure 3D.

Interaction was verified by solid-phase binding experiments. Figure 3, F and G, demonstrates a dose-dependent interaction of Cyr61 with SMTB ^1–44^ that is saturable and has calculated app *K_D_* 27.23±3.29 nM. Because PAI-1 displays high affinity and specificity for the SMTB ^1–44^ domain of VTNC [15]–[20] competition experiments were performed using a mixture containing Cyr61 and increasing concentrations of stable mutant PAI-1. Figure 3H shows that PAI-1 dose-dependently outcompetes with Cyr61 for binding to SMTB ^1–44^; 500 nM PAI-1 resulted in more than 80% inhibition. In contrast, ixolaris (500 nM) did not affect Cyr61 interaction with SMTB ^1–44^. Very similar results were obtained when PAI-1 was incubated first with SMTB ^1–44^ immobilized in the plate, followed by washing and addition of Cyr61 (not shown).

### Cyr61 Does Not Display High-Affinity Binding to SMTB ^1–44^ in Solution

It is well known that VTNC undergoes multimerization when it interacts with a number of ligands or through immobilization [9]. Therefore, it was of interest to investigate whether Cyr61 interacts with SMTB ^1–44^ in solution through isothermal titration calorimetry (ITC). As a control, binding of PAI-1 to SMTB ^1–44^ was measured by ITC with the results shown in Figure 4, A and B. Fitting of the observed enthalpies to a single-site binding model revealed a *K*_D_ of <1.0 nM for PAI-1 binding to SMTB ^1–44^. Estimation of the dissociation constant is limited by the parameter c = Ka [P], where Ka is the association equilibrium constant and [P] is the protein concentration in the calorimeter cell. Measurement of the equilibrium constant is considered unreliable when the value for c exceeds 1000 [28]. The binding reaction was exothermic, with an enthalpy change (Δ*H*) of -24 kcal/mol for binding of serpin to SMTB ^1–44^. In agreement with the results obtained by gel filtration chromatography, as well as published structural studies [15]–[20], the stoichiometry of the binding (n = 0.77±0.01) was consistent with the binding of a single PAI-1 molecule to each molecule of the SMTB ^1–44^ domain [15]–[20]. On the other hand, no observable heat was produced when SMTB ^1–44^ was titrated with Cyr61 in a similar manner (Figure 4, C and D).

### Competitive ELISA

**Figure 5:**
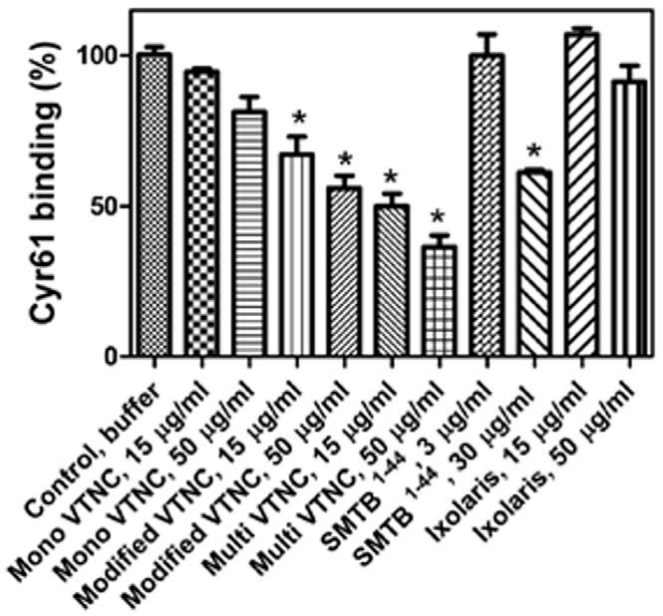
Competitive ELISA. Indicated concentrations of native monomeric VTNC, heat-modified monomeric VTNC, multimeric VTNC, SMTB ^1–44^, or ixolaris were co-incubated with a constant concentration of Cyr61 (30 nM) on microtiter wells coated with multimeric VTNC. The binding of Cyr61 to immobilized VTNC was detected with anti-Cyr monoclonal antibody followed by alkaline phosphatase-labeled anti-mouse secondary antibody and appropriate substrate as described in Materials and Methods. Results were from quadruplicate experiments.

Because ITC may not detect small heat changes that may occur in some protein interactions when studied at a given concentration [28], a second technique based on competitive ELISA [29] was employed in an attempt to estimate the relative reactivity of Cyr61 for VTNC and SMTB ^1–44^ in solution. Increasing concentrations of either monomeric VTNC, heat-modified (55°C, 1 hour) monomeric VTNC, multimeric VTNC, SMTB ^1–44^, and ixolaris (control) were co-incubated with a constant concentration of Cyr61 (30 nM) on multimeric VTNC-coated wells and assessed for ability to compete for binding of Cyr61 to immobilized VTNC. Figure 5 shows that monomeric VTNC at 50 µg/ml reacts weakly if at all with Cyr61, while heat-modified monomeric VTNC becomes reactive for Cyr61 at 15 µg/ml. On the other hand, multimeric VTNC at 15 µg/ml inhibited >50% of Cyr61 binding, while SMTB ^1–44^ produced a similar effect at high concentrations (30 µg/ml, 6 µM). Ixolaris did not affect Cyr61 interaction with immobilized VTNC.

### Cyr61 Prevents Interaction of uPAR with Vitronectin

**Figure 6:**
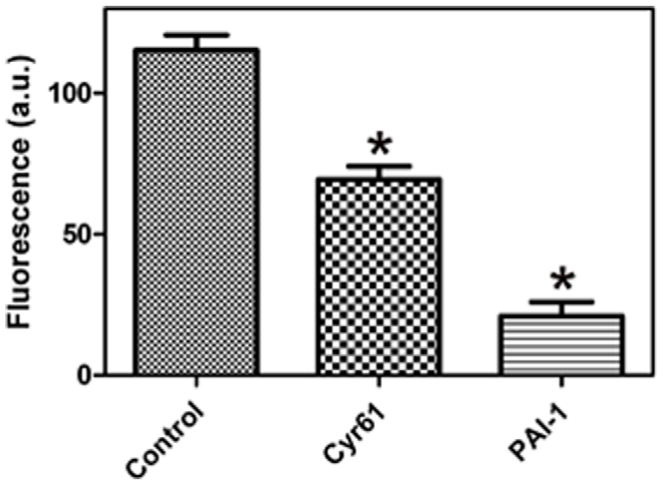
Cyr61 prevents U937 cell adhesion to vitronectin. uPAR-bearing U937 cells were incubated for 120 minutes with VTNC-coated wells previously incubated with buffer or Cyr61 or PAI-1 (1.5 µM) as described in Materials and Methods. As a control, wells were coated with gelatin only. One hundred % cell adhesion was estimated in the presence of buffer (vehicle) only, and 0% as adhesion to gelatin. Results from quintuplicate experiments are shown.

The SMTB domain mediates integrin αvβ3-, uPAR-, and PAI-1-mediated binding to VTNC [15]–[18], [30]. More precisely, the domains of SMTB ^1–44^ that interact with PAI-1 overlap extensively with those involved in the interaction with uPAR and occur independently of the integrin-recognition RGD sequence [17], [18], [31]. Accordingly, it was of interest to verify whether Cyr61 might affect uPAR interaction with VTNC, using PMA-differentiated U937 cells that reportedly express uPAR [32]–[34]. Consistent with its SMTB ^1–44^-binding properties, Cyr61 (1.5 µM) attenuates U937 adhesion to VTNC in ∼40%, while stable PAI-1 mutant, which was used as a control, blocks adhesion in >90% (Figure 6).

## Discussion

CCN family members are potent regulators of angiogenesis, chondrogenesis, and embryogenesis. More recently, novel CCN activities have been revealed, including their ability to induce apoptosis as cell adhesion substrates, to dictate the cytotoxicity of inflammatory cytokines such as TNF-α, and to promote hematopoietic stem cell self renewal [1]–[3]. Most of these activities are dependent on the association of CCN members with the cell surface and/or with the ECM. Therefore, understanding how CCN members interact with the matrix is a relevant aspect of this family of proteins. In this context, Cyr61 from 3T3 cell lysates binds to heparin-agarose beads *in vitro* [35], and CCN family members such as Cyr61/CCN1 and CTGF/CCN2 reportedly interact with heparin sulfate-containing proteoglycans (HSPG) including syndecan 4 and perlecan [36], [37]. Decorin, byglican, fibronectin, integrins, and growth factors are other molecules that have been identified as ligands for this family of proteins [1]–[3].

In this report, we demonstrate by SPR and solid-phase binding assays that Cyr61 binds with high affinity to immobilized VTNC. This discovery helps us to understand why VTNC remains associated with the ECM, being an important extracellular component with wide tissue distribution [38]. VTNC plays a significant role in a number of physiological and pathological processes involving cell adhesion and migration, angiogenesis, wound healing, and regulation of the plasminogen activation system [12], [13]. Interestingly, Cyr61 shares with VTNC a number of biologic activities, including interaction with αvβ3 integrin, promotion of attachment of HUVEC when immobilized in a plastic surface, and pro-angiogenic properties [1]–[3]. Consistent with these *in vitro* findings, Cyr61 induces angiogenesis *in vivo*, and Cyr61-null mice suffer embryonic death primarily due to vascular defects in both placenta and embryo [39]. These observations provide clear evidence for a key physiological role for the Cyr61 in angiogenesis, as has been the case for VTNC [8]. In this context, VTNC and Cyr61 have been linked to malignant progression of cancers including breast [40], ovarian [41], [42], and hepatocellular cancer [43], and both molecules are found in the lymph node [44]–[46]. Whether this association is coincidental or biologically relevant remains to be determined.

SPR and solid-phase binding assays show that Cyr61 interacts with immobilized SMTB ^1-44^ domain [15]–[20], with a similar affinity calculated for VTNC. Notably, the SMTB ^1–44^ domain has been identified as the functional region of VTNC that mediates its interaction with PAI-1, uPAR, and integrin αvβ3/αvβ5 [15]–[20], [23]. These molecules play a critical role in angiogenesis, granulation tissue formation, and wound healing. For example, αvβ3 is highly expressed in human endothelial cells and has been shown to mediate human endothelial cell migration *in vitro* and *in vivo* [9]–[11]. uPAR also contributes to matrix remodeling and cell migration through direct interaction with VTNC by focusing plasmin activation to sites of cell attachment and, in complex with integrins, can transduce intracellular signals [47]– [49]. On the other hand, PAI-1 is the primary serpin of both tissue- and urokinase-type plasminogen activators leading to attenuation of fibrinolysis and regulation of angiogenesis [12], [13], [50]. Because competition experiments confirm that PAI-1 and Cyr61 share a similar binding site in the SMTB ^1–44^ domain (Figure 3H), and as Cyr61 attenuates uPAR-mediated U937 adhesion to VTNC (Figure 6), we speculate that this interaction may affect the binding of other ligands to VTNC. In fact, the domains of SMTB ^1–44^ that interact with PAI-1 overlap extensively with those involved in the interaction with uPAR [17], [18], [31]. Structural data also indicate that the RGD sequence (residues 45–47 of the SMTB) is positioned close to PAI-1, leaving insufficient space for the RGD sequence to bind to integrins [51].

ITC experiments did not detect Cyr61 binding to the SMTB ^1–44^ domain in solution, and competitive ELISA showed that monomeric VTNC reacts weakly if at all with Cyr61; however, modified monomeric VTNC displays higher reactivity, suggesting that cryptic epitopes for Cyr61 were exposed by heat treatment as reported for VTNC interaction with specific antibodies [14], [29]. On the other hand, multimeric VTNC reacted strongly with Cyr61 in solution, possibly due to conformational changes due to self association [14]. Further, SMTB ^1–44^ effects were observed only at high concentrations (30 µg/ml; 6 µM), confirming that its interaction with Cyr61 displays low affinity that could not be estimated by ITC. These results are relevant, because *in vivo* VTNC exist in at least two distinct states and these forms have different binding properties [8]. In plasma, VTNC exists in a native, monomeric conformation that lacks exposure of conformationally sensitive epitopes. In contrast, VTNC purified from serum by heparin affinity chromatography in the presence of 8 M urea is highly multimerized and conformationally altered. Conformational change of VTNC also occurs upon binding to ligands such as heparin, thrombin-antithrombin, and complement C5b-7 or upon immobilization of VTNC in plastic surface [8], [14], [52]. Further, during tissue injury or tumor growth, VTNC is recruited from the plasma into the ECM and assumes an active conformation through multimerization [14], [38]. In other words, VTNC function is regulated by its conformation state, which has been identified as the cryptic C-terminal (heparin-binding site) [8], [14], [52] and N-terminal domains (including SMTB ^1–44^) [29]. Differences in epitope expression between native and modified VTNC have been reported before. For example, the cryptic heparin-binding site is recognized by conformationally sensitive epitope antibody 8E6, while antibodies 13H1 and 16A7 that *per se* did not react with plasma VTNC recognize VTNC treated with heparin, chaotropes, detergent, low pH, heating at 56°C, and by immobilization [14]. Moreover, modified VTNC reacted approximately 100-fold better than native VTNC with antibody MAB1244 and most notably with MAB153, an antibody directed against amino acids 1–40 of VTNC, which comprise the SMTB ^1–44^ domain [29]. The same occurs for β-endorphin binding to VTNC, which affinity is remarkably increased by immobilization [53]. Therefore, it is plausible that multimerization of VTNC creates a binding site for Cyr61 that supposedly anchors the protein to the matrix.

It is important to recognize that the preparation of Cyr61 used in this study was expressed in *Escherichia coli* and obtained from a commercial source. This may introduce a potential limitation in our study, as bacteria-expressed Cyr61 may not display functionally important glycosylation that may be necessary for activity or take part in the folding of a particular (micro)domain of the protein. While we cannot exclude this possibility, *E. coli*-expressed Cyr61 induces proliferation of NIH3T3 cells with an ED_50_ of 2–3 µg/ml (Peprotech Inc. certificate of analysis). In addition, other proteins tested as controls in this study and produced either in *E. coli* or insect cells, such as IGFBP-1, -2, and -4, did not interact with VTNC according to SPR experiments (Figure 1). Nevertheless, results presented here should be validated in the future with Cyr61 expressed by an eukaryotic expression system. Also, it cannot be excluded that Cyr61 interacts with an additional binding site in VTNC besides the SMTB ^1–44^, as recently reported for PAI-1 [21], [22]. Finally, whether Cyr61 and other CCN members associate with VTNC *in vivo* via a particular domain and whether this potential interaction has relevance to modulating the angiogenic tonus of the matrix are open questions that future studies will clarify.

## Materials and Methods

### Materials

Cyr61, IGFBP-1, -3 and -5 (expressed in *E. coli*), and IGFBP-2 and -4 (expressed in insect cells) were purchased from Peprotech (Rock Hill, NJ, USA). Native monomeric (70 kDa; product HVN) or denatured multimeric human VTNC (200 kDa; HVN-U), and PAI-1 (recombinant stable mutant form; product CPAI), were obtained from Molecular Innovations Inc. (Southfield, MI, USA). Human placenta soluble collagen Type I was from BD Biociences (Bedford, MA, USA), Horm type I fibrilar collagen was from Chrono-log (Havertown, PA, USA) and laminin was from Upstate Biotechnologies (Charlottesville, VA, USA). Anti-Cyr61 monoclonal antibody (clone 365108) was from R&D Systems (Minneapolis, MN, USA). Anti-mouse (whole molecule) alkaline phosphatase-coupled antibody developed in goat (product A3562) and *p*-nitrophenyl phosphate liquid substrate system for ELISA were purchased from Sigma Chemical Co. (St. Louis, MO, USA). Molecular weight markers, pre-cast NU-PAGE gels, and molecular biology reagents were purchased from Invitrogen (San Diego, CA, USA). Ixolaris was expressed in insect cells as described [25].

### Electrophoresis

Proteins were solubilized in LDS buffer and loaded in a 4–12% NU-PAGE gel with MES buffer using a NOVEX Powereasy500 apparatus. Proteins were stained with Coomassie Brilliant Blue G and destained in 15% methanol, 10% acetic acid. Molecular weight marker standards were: myosin (188 kDa.), BSA (62 kDa.), glutamic dehydrogenase (49 kDa.), alcohol dehydrogenase (38 kDa.), carbonic anhydrase (28 kDa.), myoglobin (18 kDa.), lysozyme (14 kDa.), aprotinin (6 kDa.) and insulin, β chain (3 kDa.).

#### Synthesis of SMTB ^1–44^, purification, refolding and mass spectrometry

SMTB ^1–44^ domain (theoretical molecular weight 5,003.54) DQESCKGRCTEGFNVD KKCQCDELCSYYQSCCTDYTAECKPQVT synthesis was carried out on a model 433A peptide synthesizer (Applied Biosystems, Inc., Fullerton, CA, USA) using solid-phase, Fmoc (9-fluorenylmethoxycarbonyl) peptide synthesis methodology with HBTU 2-(1H-benzotriazol-1-yl)-1,1,3,3-tetramethyluronium haxafluorophosphate); NMP, 1-methylpyrolidin-2-one activation. All cysteine residues were trityl protected. The Fmoc groups were removed from the N-terminal amino group of the resin-bound peptide with 20% 4-methylpiperidine in NMP. Deprotection/ cleavage from the resin was carried out with TFA/thioanisole/EDT (92∶5∶3, v/v/v) mixture at room temperature for 2.5 hours. The linear SMTB ^1–44^ was purified by RP-HPLC on a Gemini C18 (21.2 mm×250 mm) column (Phenomenex, Torrance, CA, USA) utilizing water/ acetonitrile gradient. The peptide was refolded as previously described [16]. Refolded material was purified by RP-HPLC under conditions used for the linear form. The final, folded peptide showed the correct molecular mass of 5,003.00 Da. It was biologically active based on its interaction with PAI-1 estimated by surface plasmon resonance (SPR) experiments, gel-filtration chromatography, and isothermal titration calorimetry (see Results).

### SPR Analysis

All SPR experiments were carried out in a T100 instrument (Biacore Inc., Uppsala, Sweden) following the manufacturer's instructions. The Biacore T100 evaluation software was used for kinetic analysis. Sensor CM5, amine coupling reagents, and buffers were also purchased from Biacore Inc. (Piscataway, NJ, USA). HBS-P (10 mM Hepes, pH 7.4, 150 mM NaCl, and 0.005% (v/v) P20 surfactant) was used as the running buffer for all SPR experiments, which were carried out as previously described [54]. For immobilization and kinetic analysis, monomeric (10 µg/ml) or multimeric (10 µg/ml) VTNC in acetate buffer pH 4.5 were immobilized over a CM5 sensor via amine coupling, resulting in a final immobilization of 667.4 RU and 625.2 RU, respectively. In some experiments, VTNC was immobilized at 2851.4 RU. The SMTB ^1–44^ domain (100 µg/ml in acetate buffer pH 4.5) was immobilized as above at 337.2 RU. Kinetic experiments were carried out with a contact time of 90 seconds at a flow rate of 30 µl/minute at 25°C. Cyr61-VTNC complex dissociation was monitored for 600 seconds, and the sensor surface was regenerated by a pulse of 30 seconds of 20 mM HCl at 30 µl/minute. Blank flow cells were used to subtract the buffer effect on sensorgrams. After subtraction of the contribution of bulk refractive index and nonspecific interactions with the CM5 chip surface, the individual association (*k_a_*) and dissociation (*k_d_*) rate constants were obtained by global fitting of data using the two-state reaction (conformational change) interaction model using BIAevaluation™ (Biacore, Inc.) [27]:
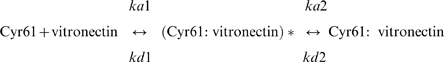



Values were then used to calculate the dissociation constant (*K_D_*)
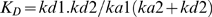



The values of average squared residual obtained were not significantly improved by fitting data to models that assumed other interactions. Conditions were chosen so that the contribution of mass transport to the observed values of *K_D_* was negligible. Also, models in the T100 evaluation software fit for mass transfer coefficient to mathematically extrapolate the true *ka* and *kd*.

### Solid-Phase Binding Assays

Monomeric or multimeric human VTNC (50 µl, both at 0.5 µg/ml in PBS, pH 7.4; 25 ng/well), or SMTB ^1–44^ (50 µl, 50 µg/ml in PBS, pH 7.4; 2.5 µg/well) were immobilized overnight at 4°C in a flat-bottom 96-well microtiter plate (Immulon 2HB; Thermo Electron Co., Milford, MA, USA). Wells were washed with 2×200 µl PBS and blocked with PBS-0.4% v/v Tween 20 (PBS-T) for 2 hours at room temperature (RT). Cyr61 (50 µl, 0–1 µM diluted in PBS-T) was added to the wells for 90 minutes. Wells were washed in PBS-T and incubated for 1 hour with anti-Cyr monoclonal antibody (100 µl, 1 µg/ml) in PBS-T. After 1 hour, wells were washed and incubated with alkaline phosphatase-coupled anti-mouse antibody (100 µl, 1∶3000 in PBS-T). After washing, 200 µl of *p*-nitrophenol phosphate liquid substrate system (Sigma-Aldrich, St. Louis, MO, USA) was added to the wells, and readings were performed at 405 nm. In all experiments, a curve containing Cyr61 (0–1 µM) was added in parallel to wells that had not been coated to estimate nonspecific binding, which was typically less than 5% of total binding at the highest Cyr61 concentration (1 µM). The app *K_d_* values for Cyr61/VTNC or Cyr61/SMTB ^1–44^ interactions were calculated by nonlinear regression analysis of the binding data according to the Langmuir isotherm equation using GraphPad Prism 5.0 (GraphPad Software, Inc., San Diego, CA, USA). Experiments were performed in quadruplicate or quintuplicate and repeated three times.

### Specificity

Human multimeric VTNC (50 µl, at 0.5 µg/ml in PBS, pH 7.4; 25 ng/well); fibrillar collagen type I Horm (50 µl, 1 µg/ml in PBS, pH 7.4; 50 ng/well), recombinant ixolaris (50 µl, 1.5 µg/ml in PBS, pH 7.4; 75 ng/well), soluble collagen type I or laminin (50 µl, 2 µg/ml in PBS, pH 7.4; 100 ng/well) were immobilized overnight as above. Wells were washed with 2×200 µl PBS-T and blocked with PBS-T for 2 hours. Then, Cyr61 (50 µl, 30 nM, in PBS-T) was added to the wells for 90 minutes. Reactions were followed as described for solid-phase binding assays.

### Competition Assays for PAI-1 and Cyr61 Binding to SMTB ^1–44^

For competition experiments, SMTB ^1–44^ (50 µl, 25 µg/ml in PBS, pH 7.4; 1.25 µg/well) was immobilized overnight, the wells were washed and blocked with PBS-T for 2 hours. In eppendorf tubes, Cyr61 (30 nM) and increasing concentrations of stable mutant PAI-1 (0, 50, 500 nM) or ixolaris (500 nM) were combined in PBS-T. Then, 50 µl of this mixture were added to SMTB ^1–44^–coated microwell plates for 90 minutes. Alternatively, PAI-1 was added to the plates for 30 minutes, washed three times, followed by addition of Cyr61 (30 nM). Cyr61 bound to SMTB ^1–44^ was estimated as described for solid-phase binding assays.

### Binding of PAI-1 to SMTB ^1–44^ by Gel Filtration

This was performed using a Pharmacia Superdex peptide PC 3.2/30 column (3.2×300 mm), 2.4 ml bed volume, assembled in Akta Purified equipment (Amersham-Pharmacia Biotech, Piscataway, NJ, USA). Column optimal separation range is 100–7000 Da, with exclusion limit 20,000 Da. Column was equilibrated in Hepes-buffered saline, pH 7.4 (HBS) at a flow rate of 40 µl/minute. For the experiments, stock solutions of PAI-1 and SMTB ^1–44^ were 55 µM and 2 mM, respectively. In an eppendorf tube, 15 or 30 µl stable mutant PAI-1 (final concentrations, 25 or 50 µM) was incubated with 1.5 µl SMTB ^1–44^ (final concentration, 100 µM) in HBS for 30 minutes. Twenty five µl was injected into the column, and formed complexes were eluted at a flow rate of 40 µl/minute and detected as peak absorption at 280 nm.

### Isothermal Titration Calorimetry

Calorimetric assays for measuring stable mutant PAI-1 or Cyr61 binding to SMTB ^1–44^ were performed using a VP-ITC microcalorimeter (Microcal, Northampton, MA, USA) at 30°C. Titration experiments were performed by making successive injections of 5 µL each of 10 µM SMTB ^1–44^ into the 1.34-mL sample cell containing 1 µM Cyr61 or PAI-1 until near-saturation was achieved. Prior to the run, the proteins were dialyzed against 20 mM Tris-HCl, 0.15 M NaCl, pH 7.4 for binding experiments. The calorimetric enthalpy (ΔH^cal^) for each injection was calculated after correction for the heat of Cyr61 dilution obtained in control experiments performed by titrating Cyr61 into buffer.

The binding isotherms were fitted according to a model for a single set of identical binding sites by nonlinear squares analysis using Microcal Origin software. The enthalpy change (Δ*H*), and stoichiometry (*n*) were determined according to equation 1:
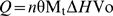
(1)where *Q* is the total heat content of the solution contained in the cell volume (Vo), at fractional saturation θ, Δ*H* is the molar heat of ligand binding, *n* is the number of sites, and M_t_ is the bulk concentration of macromolecule in Vo. The binding constant, *K*a, is described as:
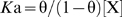
(2)where [X] is the free concentration of ligand.

The free-energy (*ΔG*) and entropy term (–T*ΔS*) of association were calculated according to:
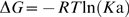
(3)

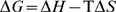
(4)


### Competitive ELISA

Interaction of Cyr61 with distinct forms of VTNC or SMTB ^1–44^ was investigated by competitive ELISA as described [29]. Briefly, microtiter wells were coated with multimeric VTNC (50 µl, 0.5 µg/ml in PBS overnight at 4°C; 25 ng/well), and nonspecific binding sites blocked with PBS-T for 2 hours at RT. Wells were co-incubated for 90 minutes with a mixture containing Cyr61 (30 nM) and different forms of VTNC or SMTB ^1–44^ (concentrations indicated in the Figure). VTNC-bound Cyr61 was estimated as described for solid-phase binding assays.

### Cell Culture

U937 (myelomonocytic cells) were obtained from American Type Culture Collection (Rockville, MD, USA) and were cultured in suspension RPMI-1640 medium (Invitrogen) containing fetal calf serum (FCS) 10% (v/v), penicillin (100 U/ml), and streptomycin (100 µg/ml) in a humidified incubator at 37°C with 5% CO_2_. One day before the experiments, U937 cells were washed and resuspended in RPMI-1640 with 10% FCS (∼300,000 cells/ml) and differentiated with PMA (50 nM) as reported [32]–[34]. Differentiated U937 cells which attached to the plastic were briefly trypsinized, washed 3 times (5 minutes, 1000×g), and resuspended in RPMI 1640 without additions (herein referred as RPMI). Cells (600,000/ml) were loaded with calcein-AM (2 µM), for 45 minutes under rotation (at RT) and washed three times. Cells were re-suspended in RPMI (no additions) to a concentration of 600,000/ml and used for cell adhesion assays as below.

### U937 Cell Adhesion to Vitronectin

Multimeric VTNC (2 µg/ml in PBS, 50 µl/well, 100 ng/well, in quintuplicate) was added to Microfluor 2 black flat-bottom high-binding 96-well plates for fluorescence applications (Thermo Electron Co., Milford, MA, USA) and immobilized overnight at 4°C. Wells were washed three times with PBS, blocked with gelatin 0.5% (w/v) for two hours, and washed with RPMI (no additions). Then, 50 µl of Cyr61 or stable mutant PAI-1 (both 1.5 µM in RPMI no additions) were added to the wells and incubated for 60 minutes at RT, followed by washing with RPMI (no additions). Fifty µl of calcein-labeled U937 cells were added to the VTNC-coated plates (30,000 cells/well) and incubated for 120 minutes at 37°C, 5% CO_2_. Plates were then inverted and washed with RPMI (no additions) three or four times, and fluorescence was measured with excitation wavelength at 490 nm and emission wavelength at 520 nm using a Spectramax Gemini XPS Microfluor plate reader (Molecular Devices, Sunnyvale, CA, USA). Fluorescence produced by cells attached to VTNC in the absence of Cyr61 or PAI-1 was set as 100% adhesion; adhesion to wells coated with gelatin only was negligible (<1% of total binding) and was set as 0% adhesion.

### Serine Protease Inhibition Assays

All assays were performed at 37°C in triplicate [55]. Cyr61 (300 nM) was pre-incubated with each enzyme tested for 10 minutes before the addition of the corresponding substrate. All enzymes used were of human origin, purified or recombinant. Thrombin, α-chymotrypsin, plasmin, and chymase were purchased from Sigma, β-tryptase was purchased from Promega (Madison, WI, USA), factor Xa was purchased from EMD Biosciences (La Jolla, CA, USA), factor XIIa was purchased from Haematologic Technologies Inc. (Essex Junction, VT, USA), kallikrein was purchased from Fitzgerald Industries International (Concord, MA, USA), elastase was purchased from Elastin Products (Owensville, MO, USA), uPA and tPA from Molecular Innovations (Southfield, MI, USA), matryptase from R&D Systems (Minneapolis, MN, USA), and sequencing-grade trypsin was purchased from Roche (Chicago, IL, USA). The amount of enzyme used in each assay and the corresponding buffer are as follows: for elastase (0.01 nM) and chymase (1.8 nM), 50 mM Hepes buffer pH 7.4, 100 mM NaCl, 0.01% Triton X-100; for trypsin (0.25 nM) and α-chymotrypsin (0.05 nM), factor XIIa (1.2 nM) and thrombin (0.01 nM), 50 mM Tris pH 8, 150 mM NaCl, 20 mM CaCl_2_, 0.01% Triton X-100; for β-tryptase (0.27 nM), 50 mM Tris pH 8, 50 mM NaCl, 0.05% Triton X-100; for kallikrein (0.8 nM), matryptase (1.2 nM), and plasmin (0.25 nM), 20 mM Tris pH 8.5, 150 mM NaCl, 0.02% triton X-100; for factor Xa (0.8 nM), 20 mM Tris pH 8, 200 mM NaCl, 5 mM CaCl_2_, 0.1%BSA; for uPA (0.7 nM) and tPA (0.06 nM), 20 mM Tris pH 8.5, 0.05% Triton X-100. The substrates used were Suc-Ala-Ala-Pro-Val-AMC for elastase, Boc-Asp-Pro-Arg-AMC for thrombin and plasmin, Boc-Gln-Ala-Arg-AMC for trypsin and uPA (Sigma), Boc-Phe-Ser-Arg-AMC for β-tryptase, Suc-Leu-Leu-Val-Tyr-AMC for chymase (Bachem, King of Prussia, PA, USA), Suc-Ala-Ala-Pro-Val-AMC for α-chymotrypsin (EMD Biosciences, La Jolla, CA, USA), methylsulfonyl-D-cyclohexylalanyl-Gly-Arg-AMC acetate for factor Xa, kallikrein, matryptase, factor XIIa, and tPA (American Diagnostica Inc., Stamford, CT, USA). All substrates were used in 250 µM final concentration in all the assays. Substrate hydrolysis rate was followed in a Spectramax Gemini XPS 96-well plate fluorescence reader (Molecular Devices) using 365 nm excitation and 450 nm emission wavelength with a cutoff at 435 nm.

### Statistical Analysis

Experiments were performed three times in quadruplicate or quintuplicate, and results are expressed as means ± SEM. Statistical significance was determined using one-way ANOVA (Bonferroni multiple post-hoc test comparison) using GraphPad Prism 5.01 software. Significance was set at P≤0.05.
